# Impact Response of the Honeycomb Sandwich Structure with Different Poisson’s Ratios

**DOI:** 10.3390/ma15196982

**Published:** 2022-10-08

**Authors:** Xiaobo Gong, Chengwei Ren, Yuhong Liu, Jian Sun, Fang Xie

**Affiliations:** 1 School of Ocean Engineering, Harbin Institute of Technology, Weihai 264209, China; 2 National Key Laboratory of Science and Technology on Advanced Composites in Special Environments, Harbin Institute of Technology, Harbin 150001, China; 3 School of Materials Science and Engineering, Harbin Institute of Technology, Weihai 264209, China

**Keywords:** honeycomb sandwich structure, Poisson’s ratios, impact resistance, finite element, dual-wall honeycomb

## Abstract

The honeycomb sandwich structure is widely used in energy-absorbing facilities because it is lightweight, has a high specific stiffness and high specific strength, and is easy to process. It also has dynamic mechanical characteristics such as a high impact resistance and high energy absorption. To explore the influence of the Poisson’s ratio on the local impact resistance, this paper compares and analyzes the local impact resistance of a series of honeycomb cores with different Poisson’s ratios under the impact of a spherical projectile at different speeds. Three typical honeycombs with negative/zero/positive Poisson ratios (re-entrant hexagon, semi-re-entrant hexagon, and hexagon) are selected to change the geometric parameters in order to have the same relative density and different Poisson ratios (−2.76–3.63). The relative magnitude of the rear face sheet displacement is in the order of negative Poisson’s ratio > zero Poisson’s ratio > positive Poisson’s ratio, which reveals that the honeycomb structure with the positive Poisson’s ratio has better protection ability than the others. Finally, a dual-wall hexagonal honeycomb is proposed. The rear face sheet displacement of the dual-wall hexagonal honeycomb sandwich structure is reduced by 34.4% at 25 m/s compared with the hexagonal honeycomb, which has a better local impact resistance.

## 1. Introduction

Because of the core layer’s compressibility and the panel’s ductility, the porous sandwich structure has excellent mechanical and physical properties and is widely used in aerospace, transportation, military industry, and construction [1,2]. The research on the mechanical behavior of porous sandwich structures under quasi-static and dynamic loads has become a hot topic [2]. Materials and structures usually used as the core layer of a sandwich structure include honeycomb materials, lattice materials, grid materials, foam materials, and thin-walled structures such as rings and tubes [3]. Among them, the honeycomb sandwich structure is widely used in energy absorption and impact-resistant facilities due to its lightweight, high specific stiffness, high specific strength, and accessible processing properties, as well as its dynamic mechanical characteristics such as its high impact resistance and high energy absorption [4,5]. Therefore, it has the potential to become an ideal impact-resistant structure [2].

Honeycomb materials offer versatility and flexible designability. With the increase in industrial demand, honeycomb materials with various topological microstructures and unique properties have been designed and processed, and different types of honeycombs will show hugely different mechanical properties [4,6]. Although the honeycomb sandwich structure has a good bending resistance, it is susceptible to impact loads. Because of the thin upper and lower panels, the panels and core layers tend to be significantly deformed and damaged when subjected to impact loads [1,6]. The honeycomb core layer mainly bears the impact energy’s damage, absorption, and dissipation. Under the impact load, the deformation mode of the honeycomb materials is complex, and elastic buckling and plastic buckling may occur [7]. Different structural forms of honeycomb materials and their sandwich structures have different deformation and failure modes. Moreover, with the change in impact load pattern and impact rate, honeycomb materials may also exhibit different compressive and energy-absorbing properties. Therefore, evaluating the bearing capacity of the structure to the impact load and the mechanical behavior of the honeycomb and its sandwich structure under the impact load has become a topic of great concern in the engineering field in recent years [2].

The mechanical properties of the honeycomb structure are affected by raw materials, topological diversity, and cell arrangement. The topological diversity makes honeycomb structures have different Poisson’s ratios, including a positive Poisson’s ratio [8], zero Poisson’s ratio [9], and negative Poisson’s ratio [10,11]. The traditional hexagonal honeycomb is a typical positive Poisson’s ratio honeycomb structure that is often used as a high-efficiency, anti-shock, and energy-absorbing structure. Its static/dynamic mechanical properties have been extensively studied. In terms of the static mechanical properties, Gibson et al. [7] firstly deduced the theoretical expressions of the mechanical property parameters such as the Poisson’s ratio, elastic modulus, and shear modulus of the honeycomb under a compressive load. Based on Gibson et al., many scholars discussed the influence of geometric parameters such as the honeycomb wall thickness, wall length, and wall inclination angle on the static platform stress and energy absorption of hexagonal honeycomb structures [12,13,14]. Ren et al. [15] summarized the expression of the mechanical property parameters and the response expression under different conditions of auxetic honeycomb structures.

In terms of the dynamic mechanical properties, researchers have studied the influence of the impact velocity and honeycomb geometric parameters on the structural in-plane deformation mode, platform stress, and energy absorption, and revealed the deformation characteristics and energy absorption mechanism of the honeycomb structure [11,16,17]. Re-entrant and semi-re-entrant hexagonal honeycombs are typically negative and zero Poisson’s ratio honeycombs, respectively [18,19]. Their static/dynamic mechanical properties have also been studied to some extent. Researchers have used theoretical analysis, numerical simulation, and experimental methods to study its equivalent elastic modulus, indentation resistance [20,21,22,23], and impact resistance [18,19], revealing that the impact resistance of honeycomb structures is affected by the cell shape, *t/l* (wall thickness/length), Poisson’s ratio, and other factors together [24,25]. In terms of the impact response of the honeycomb sandwich structure, Xie et al. [26] studied the effects of the density of the honeycomb cores, face sheet thickness, punch diameter, and impact energy on the impact loads and failure modes. Li et al. [27] explored the effects of the different impact velocities, core lengths, and wall thicknesses on the plastic stress and strain of aluminum honeycomb sandwich structures. Kamran et al. [28] developed an extended P-α model to describe the equivalent property of honeycomb materials and verified the model’s accuracy by comparison with the detailed model in terms of the ballistic limit, residual velocity, maximum displacement of the projectile, and contact time. Mohammad and Arash [29] studied the effect of weight, velocity, and energy of the projectile and the density of the foam core on the global deformation and energy loss rate of the projectile. Xue et al. [30] studied the impact resistance of the honeycomb sandwich structure under a low-speed/heavy mass load and found that the honeycomb wall thickness and cell size significantly impacted the face sheet impact force. Zhang et al. [31] discussed the contributions of total thickness, core thickness ratio, Poisson’s ratio, cell inclination angle, and blast types to the transient responses of the auxetic honeycomb sandwich plate. Nguyen [32] proposed an excellent computational approach based on polygonal meshes to comprehensively examine the free vibration, buckling, and dynamic instability behaviors of the auxetic honeycomb sandwich plate structures.

Most of the previous studies have been focused on analyzing a single-type honeycomb structure, and there is a lack of comparative analysis of the different mechanical properties of honeycomb structures with a positive/negative/zero Poisson’s ratio [20,21,33,34]. Luo et al. [35] studied the local impact resistance of honeycomb sandwich structures with different Poisson’s ratios and found that the negative Poisson’s ratio honeycomb had a more substantial impact bearing capacity, while the zero Poisson’s ratio honeycomb had a better damping and energy absorption capacity. However, the above analysis obtained different Poisson’s ratios by changing the geometric parameters, only considering the effect of the Poisson’s ratio on the impact resistance of honeycomb structures, but ignoring the effect of geometric parameters on the relative density. The relative density of the honeycomb structure directly affects its mechanical properties, such as weight and elastic modulus, crush stress, and compaction strain. It is closely related to the impact resistance of the structure, which is the critical parameter for the honeycomb structure to achieve lightweight and superior impact resistance [26].

In this paper, three typical honeycomb topologies with a positive/negative/zero Poisson’s ratio (convex, re-entrant, and semi-re-entrant cells) are selected to explore the effect of the Poisson’s ratio of the honeycomb structure on its impact resistance. To eliminate the interference of relative density, the honeycomb structure had different Poisson’s ratios (−2.76–3.63) and the same relative density by changing the geometric parameters. A comparative study of the local impact resistance of honeycomb sandwich structures with different Poisson’s ratios is numerically analyzed, which will provide engineering reference for the subsequent application of honeycomb sandwich structures. Furthermore, a novel dual-wall hexagonal honeycomb with low stiffness is proposed. The rear face sheet displacement of the novel dual-wall hexagonal honeycomb sandwich structure is significantly lower than that of the hexagonal honeycomb, and it has a better local impact resistance.

## 2. Materials and Methods

### 2.1. Topological Diversity

**Figure 1:**
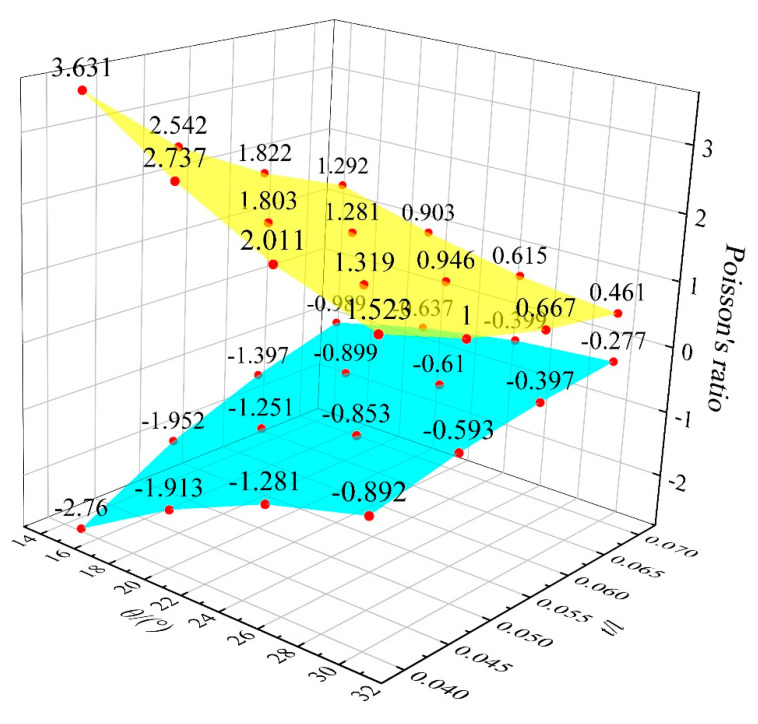
The Poisson’s ratio of the honeycomb structure with different geometries.

The Poisson’s ratio of the hexagonal honeycomb core is directly affected by the cell topology (i.e., convex honeycomb with positive Poisson’s ratio, semi-re-entrant honeycomb with zero Poisson’s ratio, and re-entrant honeycomb with negative Poisson’s ratio). More specifically, the Poisson’s ratio of different topologies can be calculated by the equation shown in Table 1, which is derived based on standard beam theory, including an inclined angle *θ* and struts ratio *t/l*. By changing the geometric parameters such as *h*, *l*, *θ*, and t, the relative density is almost the same (0.0613) and the Poisson’s ratios are different (−2.76–3.63). The *t/l* value range of the honeycomb structure is 0.04–0.07, the interval is 0.01, the value of *θ* is 15–30°, and the interval is 5°. According to the equation in Table 1, the Poisson’s ratio of the honeycomb sandwich structure with different geometries is shown in Figure 1. The result reveals that for convex and re-entrant honeycombs, lower *θ* or *t/l* gives rise to a larger absolute value for the Poisson’s ratio. From the cell topology, it can also be observed that the overall structure becomes more slender and thinner with a decrease in *θ* or *t/l*. Therefore, generating a large transverse strain under a certain longitudinal strain is easier, which leads to a higher absolute value of the Poisson’s ratio. The Poisson’s ratio of the semi-re-entrant honeycomb is independent of the geometry parameters and is only determined by the cell topology, which is always zero.

### 2.2. Finite Element Model

**Figure 2:**
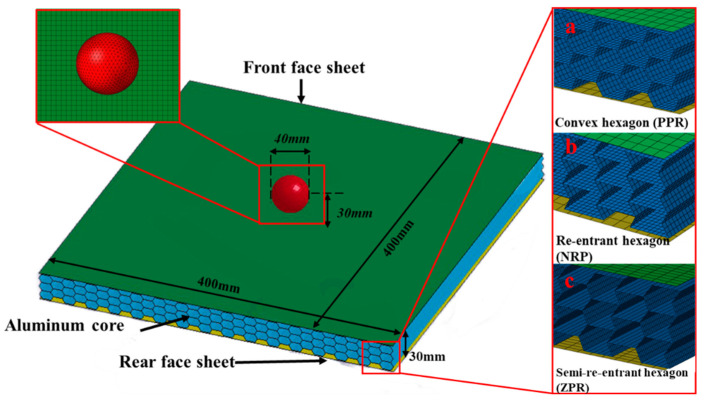
Finite element model of three types of honeycomb structures: (**a**) convex hexagon honeycomb with positive Poisson’s ratio; (**b**) re-entrant hexagon honeycomb with negative Poisson’s ratio; (**c**) semi-re-entrant hexagon honeycomb with zero Poisson’s ratio.

The honeycomb sandwich structures were modeled, including a front face sheet, rear face sheet, and honeycomb cores, as shown in Figure 2. The dimensions were 400 mm × 400 mm × 30 mm, and the front and rear face sheets were 1 mm thick. The 40 mm diameter projectile impacted the center of the honeycomb sandwich face sheet at initial speeds of 25 m/s, 50 m/s, and 100 m/s. For honeycomb sandwich structures to effectively dissipate the energy of a projectile, the projectile’s diameter should be larger than the size of the cells [36]. The projectile will penetrate through the core for projectile diameters less than the cell size. At the same time, considering the requirements of the experimental equipment, a 40 mm spherical projectile was selected in this paper. When the projectile hit the panel, its affected area of the honeycomb panel was proportional to the projectile’s diameter. When the panel size was significantly larger than the projectile size, the panel size did not affect the simulation results. When the projectile diameter was 60 mm and the panel size was 500 × 500 mm, a good simulation effect was achieved [35], and the ratio of the projectile diameter to the panel size was 3/25. Therefore, this paper selected a square panel of 400 × 400 mm. At this time, the percentage of the projectile diameter to the panel size was 1/10, a relatively large number.

The LS-DYNA V 971 software (ANSYS, Inc., Canonsburg, PA, USA) was used to analyze the impact resistance of the honeycomb structure. The honeycomb sandwich structure was meshed by a four-node shell163 element with a size of 1 mm. The projectile was meshed by an eight-node solid164 with a mesh size of 1 mm. The rear face sheet boundary condition was simply supported on all sides. To avoid self-penetration, the contact between the projectile and face sheet, and core and face sheet, were set as automatic surface-to-surface contact. The projectile was set as 45 steel using the *PIECEWISE_LINEAR_PLASTICITY constitutive model with a density of 7.8 × 10^3^ kg/m^3^, an elastic modulus of 210 GPa, a Poisson’s ratio of 0.3, and yield stress of 355 MPa. The face sheet and honeycomb core material was aluminum with a density of 2.7 × 10^3^ kg/m^3^, an elastic modulus of 68 GPa, a Poisson’s ratio of 0.3, and yield stress of 130 MPa. The MATSUM and RCFORC commands were used to output the energy and impact force of the structure, respectively. The duration of the analysis process was 4 ms, at which time the projectile would have completed the impact process and rebounded. The time step was determined by LS-DYNA V 971 software according to *Δt* = 0.9 *l/c*, where *l* is the characteristic length, *c* is the propagation speed of the wave, and the time step scaling factor is 0.9.

### 2.3. Model Validation

**Figure 3:**
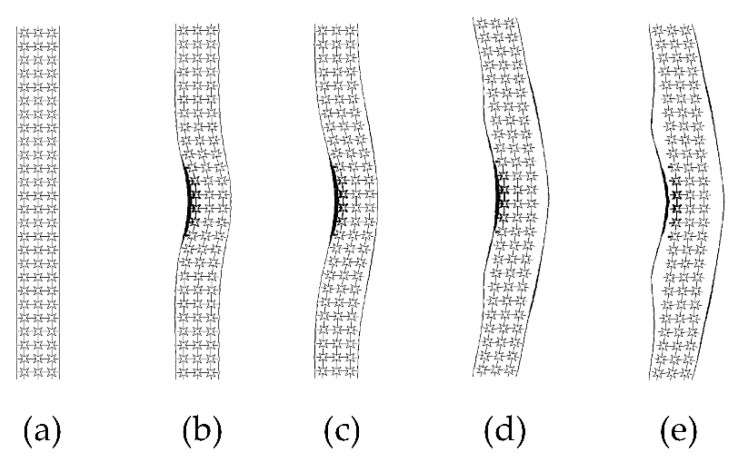
Impact simulation results of the star-shaped honeycomb structure: (**a**) 0 ms; (**b**) 0.25 ms; (**c**) 0.5 ms; (**d**) 5 ms; (**e**) 6 ms.

The comparison with the similar conditions of a previous work by Gao [37] verified the numerical simulation used in this paper. The final deflection of the sandwich structure’s rear face sheet center was simulated with LS-DYNA V 971 software (ANSYS, Inc., Canonsburg, PA, USA). The star honeycomb has a negative Poisson’s ratio, and the Poisson’s ratio value was 0.44. The projectile material was foam aluminum using the ** MAT_DESHPANDE_FLECK_FOAM constitutive model with a density of 2.54 × 10^3^ kg/m^3^ and an elastic modulus of 210 GPa. The face sheet and honeycomb core were both AlSi_10_Mg using the * PIECEWISE_LIN--EAR_PLASTICITY constitutive model with a density of 2.7 × 10^3^ kg/m^3^, an elastic modulus of 56 GPa, a Poisson’s ratio of 0.3, and a yield stress of 180 MPa. This paper compared the deformation and the final deflection of the rear face sheet of the honeycomb structure under an impact load of 202.2 m/s. Figure 3 shows the deformation of the honeycomb sandwich structure in the impact process, which is in good agreement with the experimental results [37]. The specific data comparison is shown in Table 2. The deflection error of the rearing plate of the sandwich structure is only 1%.

## 3. Results

### 3.1. Front Face Sheet Impact Force

**Figure 4:**
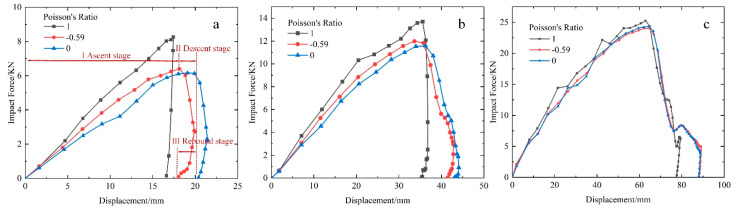
Face sheet impact forces vs. displacement of honeycomb sandwich structures with different Poisson’s ratios: (**a**) 25 m/s; (**b**) 50 m/s; (**c**) 100 m/s.

The impact force displacement of the honeycomb sandwich front face sheet under the different impact speeds is shown in Figure 4. The deformation modes of the sandwich face sheets include local indentation, global deflection, and elastic unloading (rebound), which correspond to three distinct response stages on the impact force–displacement curves: ascent, descent (softening), and rebound stage, respectively. The first stage is the local indentation stage; the load is mainly carried by the front face sheet and the honeycomb core compression. Therefore, the impact force of the ascent stage gradually increases, which absorbs most of the energy during the impact process. The second stage is the global deflection stage, defined as the horizontal distance between the displacement when the impact force is maximum and the maximum displacement of the front face sheet. At this stage, the load is mainly carried by the overall deflection of the structure and a softening stage with a decreasing impact force would have occurred. The third stage is related to the elastic strain energy released by the honeycomb structure.

**Figure 5:**
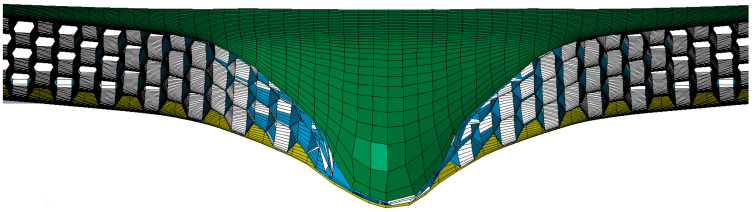
Rebound stage of the honeycomb sandwich structure with a Poisson’s ratio of 1.

The front face sheet impact force of the honeycomb sandwich structure is convex > re-entrant > semi-re-entrant under a 25 m/s and 50 m/s impact load. When impacted at a low speed, the honeycomb core in the impact compression zone is not sufficiently densified, and the vertical (impact direction) walls mainly absorb the impact energy [7,16]. Under the condition of the same relative density, the convex honeycomb has the largest number of honeycomb walls in the vertical direction, which is easier to absorb the impact energy of the projectile and minimize the displacement of the rear face sheet. However, the shrinkage elasticity of the convex honeycomb structure was poor with the shortest softening stage and rebound stage. Semi-re-entrant honeycombs had the slightest impact but the longest platform stage, which means that they had a good cushioning performance. Under 100 m/s impact speed, all honeycomb structures produced more plastic deformation. The rebound stages of honeycomb sandwich structures became shorter, and the maximum face sheet impact force came closer. This indicates that the Poisson’s ratio had little influence on the impact resistance of honeycomb structures under a high-speed impact load. Furthermore, the impact force produces a sudden increase before the honeycomb structure rebounds, as shown in Figure 4c. This is because the honeycomb core layer was entirely compressed and densified under a relatively high-speed impact, as shown in Figure 5, resulting in a stronger impact force.

### 3.2. Displacement of the Rear Face Sheet

In general, the maximum displacement of the front face sheet, the maximum displacement of the rear face sheet, and the difference between them were defined as the dent depth, deflection, and core compression depth, respectively. The deflection reflected the impact intensity of the rear face sheet, and the smaller deflection predicted a better buffering performance. The deformation capacity of the structures is generally related to the core stiffness. It can be seen from the elastic modulus formula of a honeycomb structure that the higher *t/l* and the smaller *θ* would lead to a higher stiffness.

The rear face sheet displacement of the honeycomb sandwich structure at different impact speeds was given in Table 3. For convex and semi-re-entrant honeycombs under 25 m/s and 50 m/s impact speeds, the deformation ability of the honeycomb core decreased with the increased stiffness, which weakened the pressing effect of the honeycomb core on the rear face sheet and reduced the displacement of the rear panel. For re-entrant honeycombs, when the negative Poisson’s ratio honeycomb was subjected to an impact load, the material gathered to the impact area and became denser, resulting in an increase in stiffness, which in turn led to a decrease in the deformation of the honeycomb core and an increase in the rear face sheet displacement. Therefore, under the same impact conditions, the negative Poisson’s ratio honeycomb sandwich structure had the largest rear face sheet deflection. For convex and semi-re-entrant honeycombs under 100 m/s impact speeds, the honeycomb core in the contact area was prone to full densification and loss of deformability, which caused a large displacement in the rear face sheet. Therefore, the honeycomb structure needed a higher stiffness to prevent the core layer from entering the dense stage too early. For re-entrant honeycombs, it is necessary to reduce the wall thickness to prevent the structure from entering the dense stage prematurely, which gives the honeycomb core more deformable space.

**Figure 6:**
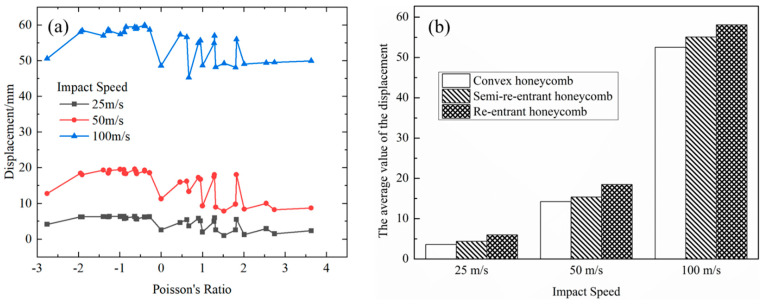
The rear face sheet displacement of the honeycomb sandwich structure with different Poisson’s ratios: (**a**) displacement; (**b**) the average value of displacement.

Figure 6 further compares the rear face sheet displacements of the honeycomb sandwich structures with different Poisson’s ratios. Under the same impact speed, a negative Poisson’s ratio honeycomb showed a large displacement and small fluctuation because of its dynamic stiffness enhancement and minimum densification strain threshold; a zero Poisson’s ratio honeycomb showed a small displacement; a positive Poisson’s ratio honeycomb showed a fluctuation in displacement due to the inconsistency of the Poisson’s ratio and *t/l*. The relative magnitude of the rear face sheet displacement was in the order of negative Poisson’s ratio > zero Poisson’s ratio > positive Poisson’s ratio, which is the result of the joint influence of the honeycomb structure stiffness and *t/l* (stiffness mainly affects the zero and positive Poisson’s ratio honeycomb, and *t/l* mainly affects the negative Poisson’s ratio honeycomb). Therefore, it is essential to reduce the rear face sheet displacement to find the appropriate stiffness.

### 3.3. Energy Absorption

**Figure 7:**
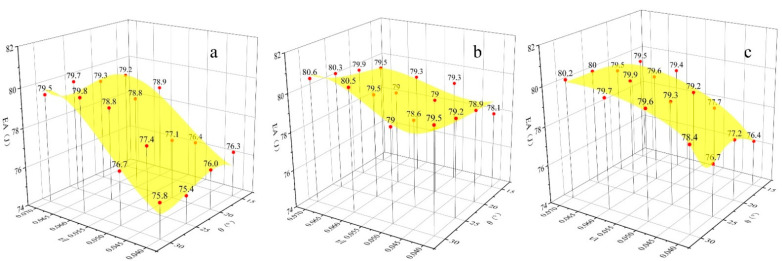
Energy absorption of the honeycomb structure with different Poisson’s ratios at 25 m/s impact speed: (**a**) Poisson’s ratio is positive; (**b**) Poisson’s ratio is negative; (**c**): Poisson’s ratio is zero.

**Figure 8:**
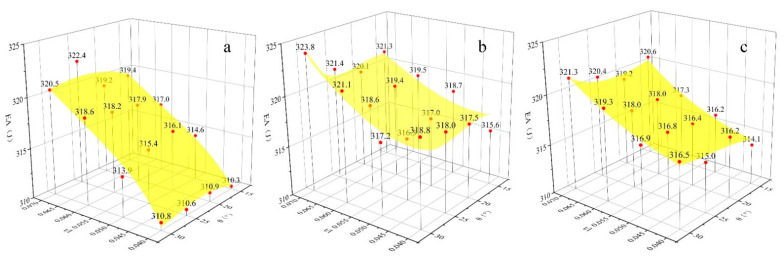
Energy absorption of the honeycomb structure with different Poisson’s ratios at 50 m/s impact speed: (**a**) Poisson’s ratio is positive; (**b**) Poisson’s ratio is negative; (**c**): Poisson’s ratio is zero.

**Figure 9:**
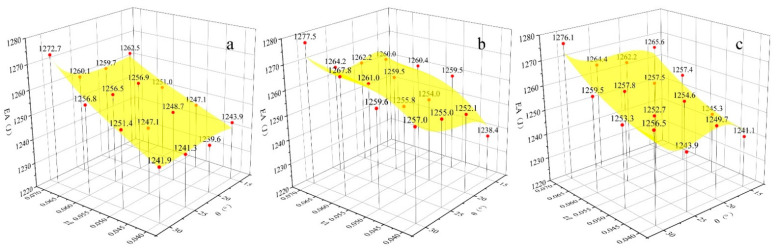
Energy absorption of the honeycomb structure with different Poisson’s ratios at 100 m/s impact speed: (**a**) Poisson’s ratio is positive; (**b**) Poisson’s ratio is negative; (**c**): Poisson’s ratio is zero.

**Figure 10:**
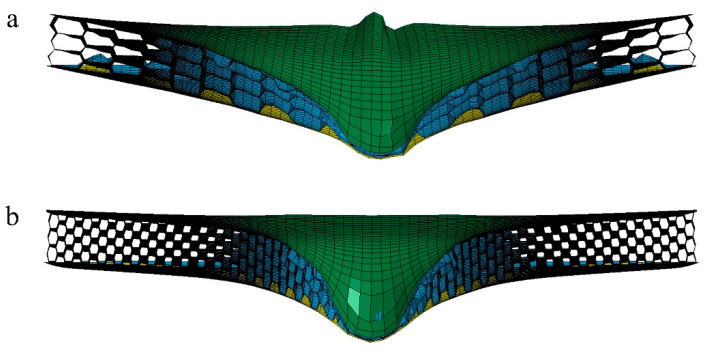
Deformation of the honeycomb structure with different geometric parameters under 100 m/s impact speed: (**a**) *t/l* = 0.07, *θ* = 30°; (**b**) *t/l* = 0.04, *θ* = 15°.

The energy absorption of the honeycomb sandwich structure under different impact speeds is demonstrated in Figure 7, Figure 8 and Figure 9. The total energy absorption was significantly positively correlated with the value of *t/l*, i.e., the energy absorption increased as *t/l* increased. This is because *t/l* is positively related to the honeycomb stiffness and the structure produces greater global plastic deformation after an impact, which will improve the energy absorption of the honeycomb. However, there is no clear correlation between the energy absorption and the Poisson’s ratio. Under 25 m/s and 50 m/s impact speeds, the honeycomb core is not fully densified and the energy absorption is mainly determined by the number of honeycomb cells. The higher *t/l* means a smaller number of honeycomb cells, which will increase the honeycomb core’s plastic deformation threshold and energy absorption. The change in *t/l* and *θ* had a smaller effect on the energy absorption of the re-entrant honeycomb. This is because the disadvantage of the smaller energy absorption of the re-entrant honeycomb with smaller *t/l* and *θ* was compensated by the stronger effect of the negative Poisson’s ratio. Under 100 m/s impact speeds, the energy absorption difference between the three honeycomb structures was very small due to the fully dense honeycomb core. The deformation of the honeycomb structure with two different geometric parameters is given in Figure 10, which shows the fully dense state. Therefore, the honeycomb sandwich structure with large *t/l* and *θ* shows a strong energy absorption ability. It is worth noting that the re-entry honeycomb with smaller *t/l* and *θ* also has a strong energy absorption capacity, but at this time, the displacement of the rear face sheet is larger, sacrificing part of the protective performance. The rear face sheet displacement and energy absorption must be fully considered in the impact-resistant design of the honeycomb structure.

## 4. Dual-Wall Hexagonal Honeycomb Structure

### 4.1. Structural Parameters

**Figure 11:**
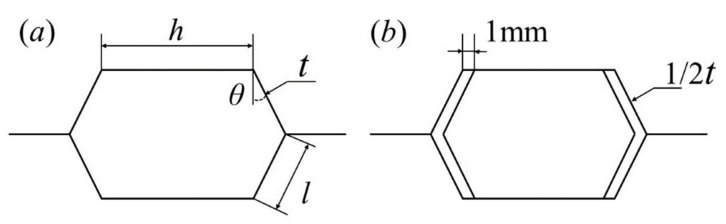
Convex honeycomb and dual-wall hexagonal honeycomb: (**a**) convex honeycomb; (**b**) dual-wall hexagonal honeycomb.

The results of Section 3.2 show that the magnitude order of the rear face sheet displacement is negative Poisson’s ratio > zero Poisson’s ratio > positive Poisson’s ratio, so the positive Poisson’s ratio honeycomb structure has the best protection ability. The displacement of the rear face sheet is significantly affected by the structural stiffness. As the stiffness becomes smaller, the deformation ability of the honeycomb core becomes stronger and the extrusion to the rear face sheet becomes weaker. To further reduce the displacement of the rear face sheet, a dual-wall hexagonal honeycomb structure with double walls with a half thickness of the single wall is proposed in order to reduce the stiffness and enhance the honeycomb core’s deformability, as shown in Figure 11.

### 4.2. Rear Face Sheet Displacement of the Dual-Wall Hexagonal Honeycomb Sandwich Structure

**Figure 12:**
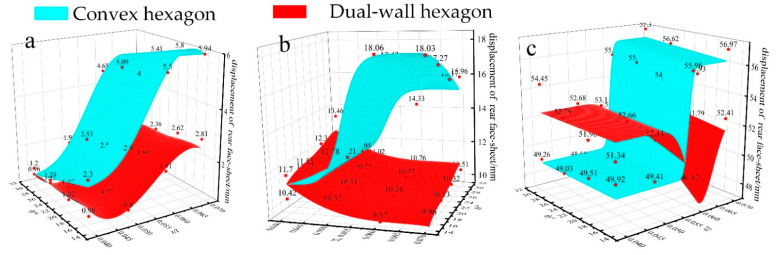
The rear face sheet displacement of the honeycomb sandwich structures at different impact speeds: (**a**) 25 m/s; (**b**) 50 m/s; (**c**) 100 m/s.

The impact resistance of the dual-wall hexagonal honeycomb sandwich structure that withstands local load by a spherical projectile impact is studied in this section. The rear face sheet displacement of the different honeycomb sandwich structures is given in Figure 12. Similar to the convex honeycomb structure, the deformation ability of the dual-wall hexagon honeycomb core decreases with the increased stiffness, which weakens the pressing effect of the honeycomb core on the rear face sheet and reduces the displacement of the rear face sheet.

**Figure 13:**
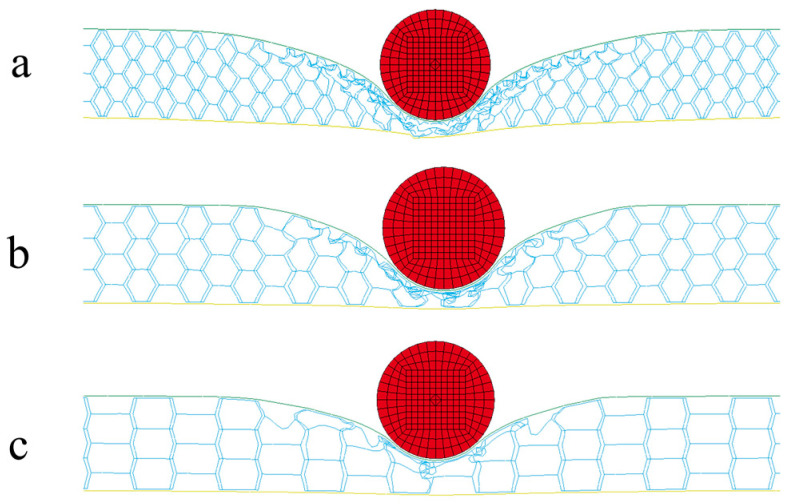
Deformation of the dual-wall honeycomb structure: (**a**) *t/l* = 0.04, *θ* = 30°; (**b**) *t/l* = 0.05, *θ* = 30°; (**c**) *t/l* = 0.07, *θ* = 15°.

Intuitively, when the impact speed is 25 m/s and 50 m/s, the rear face sheet displacement of the dual-wall honeycomb sandwich structure is smaller than that of the convex honeycomb structure, and when the impact speed is 100 m/s, there is no obvious magnitude. Under 25 m/s impact speed, the dual-wall hexagonal honeycomb presents an excellent cushioning capability and the rear face sheet displacement of the sandwich structure is less than 3 mm, which is only 30–50% of the convex honeycomb. The rear face sheet displacement obtains the minimum value of 0.63 mm with *t/l* = 0.05 and *θ* = 30°, which is 34.4% smaller than that of the convex honeycomb structure. The deformation of the dual-wall honeycomb structure with a different geometry is presented in Figure 13, which shows that the densification is introduced into the honeycomb core in advance with little deformation resistance due to its low stiffness. Because of the enhanced inertial effect, the displacement reduction effect gradually weakens with the increase in the impact velocity. Under 50 m/s impact speed, the rear face sheet displacement of the dual-wall hexagon honeycomb structure obtains the minimum value of 9.57 mm with *t/l* = 0.06 and *θ* = 15°, which is 11.6% smaller than that of the convex honeycomb structure. Under 100 m/s impact speed, the rear face sheet displacement is not always smaller than that of a convex hexagon honeycomb. When the *t/l* is large, the dual wall hexagon presents a smaller rear face sheet displacement, while when *t/l* is small, the hexagon is larger. For the dual-wall hexagonal honeycomb with smaller *t/l* (*t/l* < 0.06), which is fully densified under the impact of a spherical projectile due to its small stiffness, the honeycomb core stiffness is too small to resist deformation under the high-speed impact, so the rear face sheet displacement is larger than that of the convex honeycomb structure.

## 5. Conclusions

This paper presents a numerical analysis of the localized impact resistance of three typical honeycomb topologies with positive/negative/zero Poisson’s ratios (hexagon, concave hexagon, and half-concave hexagon). The selected honeycomb structures have the same relative density and different Poisson’s ratios. The influence of the Poisson’s ratio on the impact resistance of honeycomb structures is compared and analyzed.

When the impact velocity is low, the impact force of the front face sheet is hexagon > concave hexagon > half concave hexagon. When it is high, the impact force of the front face sheet is determined by *t/l* and has nothing to do with the Poisson’s ratio. The relative magnitude of the rear face sheet displacement of the honeycomb structure is negative Poisson’s ratio > zero Poisson’s ratio > positive Poisson’s ratio, indicating that the positive Poisson’s ratio honeycomb structure has a better local impact protection ability than the others. For energy absorption, the relationship with the Poisson’s ratio is not obvious, but it is related to the *t/l* and *θ* of the honeycomb. In general, larger *t/l* and *θ* increase the plastic deformation of the honeycomb structure and thus improve the energy absorption. It should be noted that the energy absorption and the displacement of the rear face sheet are often in conflict. The honeycomb sandwich structure with a strong deformation capacity of the core improves the energy absorption capacity, but the displacement of the rear face sheet is larger. Therefore, the rear face sheet displacement and energy absorption need to be compromised when designing the impact resistance of the honeycomb structure.

To further reduce the displacement of the rear panel of the honeycomb sandwich panel and to improve the protection ability of the honeycomb sandwich structure, a dual-wall hexagon honeycomb structure is proposed. The rear face sheet’s displacement of the dual-wall hexagon honeycomb is only 30–50% of the convex hexagon honeycomb, which provides a reference for the design of the impact protection structure.

## Figures and Tables

**Table 1:** Poisson’s ratio of three different honeycomb structures.

Honeycomb Structure		Poisson’s Ratio
convex	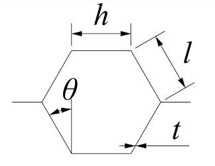	$v_{12}=\frac{\cos^{2}\theta }{(h/l+\sin\theta )\sin\theta }$
re-entrant	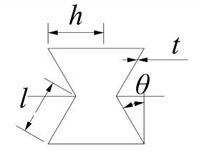	$v_{12}=-\frac{\cos^{2}\theta }{(h/l-\sin\theta )\sin\theta }$
semi-re-entrant	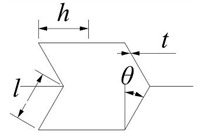	0

**Table 2:** Final deflection of the rear face sheet center of the star-shaped sandwich structure.

Impact Speed (m/s)	Final Deflection of the Rear Face Sheet Center of the Sandwich Structure (mm)		
	Experimental Result	Simulation Result	Error
202.2	20.5	20.3	1%

**Table 3:** The displacement of the honeycomb sandwich structure rear face sheet at different impact speeds.

The Displacement of the Rear Face Sheet (mm)		Convex Honeycomb			Semi-Re-Entrant Honeycomb			Re-Entrant Honeycomb		
*t/l*	*θ* (°)	25 m/s	50 m/s	100 m/s	25 m/s	50 m/s	100 m/s	25 m/s	50 m/s	100 m/s
0.04	15	2.30	11.70	49.92	4.11	12.16	50.77	4.11	12.72	50.56
	20	1.48	11.23	49.51	2.61	11.84	50.03	6.21	18.02	58.47
	25	1.23	11.09	49.03	2.63	11.27	50.15	6.22	18.46	58.66
	30	0.96	10.82	49.26	2.54	11.97	49.31	5.76	18.39	57.92
0.05	15	2.90	12.78	49.41	3.54	12.89	48.56	6.20	18.46	57.98
	20	2.54	12.21	48.09	4.44	16.28	57.79	6.34	19.27	58.28
	25	2.53	11.95	48.20	4.48	16.20	56.69	5.87	18.33	59.46
	30	1.95	12.30	48.68	4.68	15.53	58.59	5.57	18.29	59.19
0.06	15	5.50	18.06	55.96	4.78	16.84	57.12	6.28	19.32	56.99
	20	4.96	17.43	54.91	4.55	16.70	56.99	6.34	19.44	58.03
	25	5.09	16.75	55.63	4.88	16.68	58.34	5.73	18.94	58.87
	30	4.65	14.33	55.75	4.77	17.10	58.33	6.12	19.32	59.70
0.07	15	5.94	18.03	56.97	5.82	18.29	57.08	6.31	19.50	57.39
	20	5.80	17.27	54.93	5.44	17.95	56.94	6.32	19.58	59.40
	25	5.41	16.17	56.62	5.56	17.58	56.23	6.20	19.05	59.97
	30	4.63	15.96	57.30	5.25	16.53	58.44	6.24	18.57	58.64

## Data Availability

Not applicable.
